# Putting measurement on a diet: development of a core set of indicators for quality improvement in the ICU using a Delphi method

**DOI:** 10.1186/s12913-022-08236-3

**Published:** 2022-07-05

**Authors:** Marieke Zegers, Rutger Verhage, Gijs Hesselink, Johannes G. van der Hoeven

**Affiliations:** 1 Department of Intensive Care Medicine, Radboud University Medical Center, Nijmegen, The Netherlands; 2 Intensive Care Medicine Radboudumc, Internal Postal Code: 707, P.O. Box 9101, 6500 HB Nijmegen, The Netherlands

**Keywords:** Consensus methods, Intensive care, Quality indicators, Quality improvement, Governance

## Abstract

**Background:**

The number and efficacy of indicators used to monitor and improve the quality of care in Intensive Care Units (ICU) is debatable. This study aimed to select a consensus-based core set of indicators for effective quality improvement in the ICU.

**Methods:**

A Delphi study with a panel of intensivists, ICU nurses, and former ICU patients or relatives (*n* = 34) from general, teaching, and academic hospitals. Panelists completed a questionnaire in which they scored 69 preselected quality indicators on relevance using a nine-point Likert scale. Indicators were categorized using the rated relevance score into: ‘accepted, ‘equivocal’ and ‘excluded’. Questionnaire results were discussed in focus groups to reach consensus on the final set.

**Results:**

Response rates for the questionnaire and focus groups were 100 and 68%, respectively. Consensus was reached on a final set of 17 quality indicators including patient reported outcome measures (PROMs) and patient reported experience measures (PREMs). Other quality indicators relate to the organization and outcome of ICU care, including safety culture, ICU standardized mortality ratio, and the process indicator ‘learning from and improving after serious incidents’.

**Conclusions:**

ICU clinicians and former patients and relatives developed a consensus-based core set of ICU quality indicators that is relatively short but comprehensive and particularly tailored to end-users needs.

**Supplementary Information:**

The online version contains supplementary material available at 10.1186/s12913-022-08236-3.

## Background

In healthcare, quality measurement is paramount for improvement. Quality measurement is often based on the use of quality indicators; i.e., standardized (quantitative and qualitative) measures used to determine and track clinical performance and outcomes [1]. However, the large number of quality indicators for which clinicians need to record data (i.e., quality registrations) diverts time from real quality improvement in clinical practice and even worse, diverts time from providing actual patient care [2]. In Dutch ICUs, clinicians spend 52 min per shift on quality registrations. Overall, 57% of these registrations are primarily performed for accountability purposes, 19% for institutional governance and 25% for quality improvement objectives [3]. Besides the time spend on quality registrations, their efficacy is also under debate. There is little evidence that the considerable effort and resources invested by clinicians in these registrations, including those used for benchmarking, lead to improved outcomes [4].

The inefficient nature and large amount of quality registrations frustrates hospital physicians and nurses. Registrations that are perceived as unreasonable by health care providers are negatively associated with their intrinsic motivation [3]. Also, more time spent on administrative tasks increases the risk of burnout [5]. Paradoxically, the large number of quality registrations prevents patients from receiving timely and appropriate care [2, 3]. Therefore, measuring only what really matters and mainly for continuous learning is key to increase the effectiveness of registrations for quality improvement [6]. Using a core set of quality indicators could help to prevent valuable time and resources being wasted on generating information that isn’t likely to improve care quality or isn’t used at all [2, 3]. Developing such a core set by those who are primarily involved in the delivery and receipt of care is important to ensure that registrations have value, registration tasks are supported by clinicians in practice, and that information is used to improve patient outcomes and experiences.

Several core sets of outcomes for critical care settings have been developed previously for critically ill patients (e.g., patients with traumatic brain injury, acute respiratory failure patients). However, these sets were not developed with the input of patients [7, 8], were not systematically developed with expert consensus [9], or were aimed to provide key outcomes for clinical research [10–12]. Therefore, the aim of this study was to establish a stakeholder consensus-based core set of quality indicators for effective quality improvement in the ICU.

## Methods

### Study design, setting and participants

**Fig. 1:**
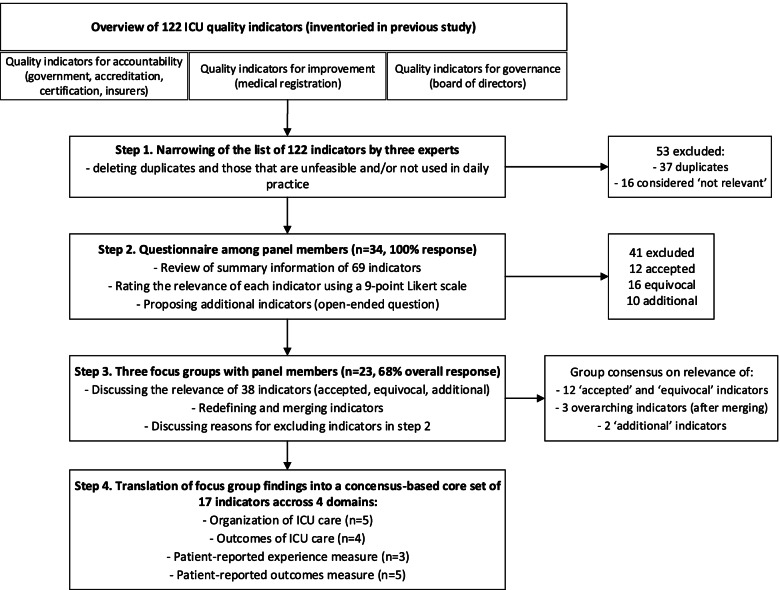
Flow diagram outlining the development of the core set of ICU quality indicators. ICU = Intensive Care Unit

We performed a Delphi study, consisting of four steps, to select a core set of quality indicators (Fig. 1). The Delphi technique is an iterative multistage process, designed to transform expert opinions into group consensus [13]. The Delphi study was performed in the period between April and July of 2017 with a panel of experts: intensivists and ICU nurses from one academic, one tertiary teaching and two general Dutch hospitals, and former ICU patients and relatives. Clinicians were recruited by the authors and selected based on their involvement in quality improvement in their ICU. Former patients and relatives were recruited via the FCIC (Family and Patient Centered Intensive Care; www.fcic.nl), the Dutch foundation for former ICU patients and relatives. Other stakeholders such as hospital managers, quality officers and representatives of the health inspectorate and accreditation agencies were excluded from the panel to ensure that this core set is solely valued by those who are primarily involved in the receipt and delivery of ICU care.

Ethical approval was sought from the local Ethics Committee ‘CMO region Arnhem-Nijmegen’ (registration number: 2017/3247). The committee judged that ethical approval was not required under Dutch National Law. All participants received written information about the project and its aims and gave verbal informed consent.

### Step 1. Narrowing of a pre-existing list of ICU quality indicators

To select a core set of quality indicators, an overview of all indicators pertaining to the ICU is necessary. In a previous study, we already inventoried all quality indicators that are used in the Dutch ICUs for quality improvement, governance, and accountability [14]. Both quantitative and qualitative measures were included, such as mortality rate and narratives of former ICU patients and their relatives describing their experiences with ICU care, respectively.

The inventory resulted in a total of 122 quality indicators [14]. One intensivist and expert in quality improvement in the ICU (RV) and one senior researcher specialized in quality improvement (MZ) reduced the list of 122 indicators by removing duplications (those that were registered twice for separate stakeholders, with overlapping definitions) and quality indicators very likely to be considered ‘not relevant’ (e.g., length of mechanical ventilation of patient under the age of 18). Indicators were subsequently categorized into overarching domains. Disagreement about removing, including, and categorizing indicators were resolved by discussion between RV and MZ, and if needed, a professor in critical care and opinion leader in the field of intensive care medicine (HH) was consulted to facilitate decisions.

### Step 2. Questionnaire

In step 2, we asked panel members to rate the relevance of each of the 69 quality indicators using a paper-based survey. Panel members were specifically instructed to rate each indicator on a nine-point Likert scale ranging from 1 (not at all relevant) to 9 (very relevant) by asking: ‘’Please rate on a scale from 1 to 9 to what extent the indicator and related measurement data is useful for improving clinical performance, health outcomes and patient experiences ‘’. An operationalization of each indicator assisted the panel members in the rating process. The questionnaire ended with an open-ended question: ‘’What quality indicators are relevant to improve patient care and outcomes, but were not mentioned in the questionnaire’’?

After fulfilling the questionnaire, the indicators were divided into three categories [14–16], based on the rated relevance scores by panelists:

- *Accepted*
  A convincing majority of the panel members considered the indicator relevant: at least 70% of participants scored 7, 8 or 9 and the median was at least 8.
- *Equivocal*
  Extremely skewed distribution: at least 30% of the panel members scored 1, 2 or 3 and at least 30% of participants scored 7, 8 or 9; or somewhat skewed distribution: at least 70% of the panel members scored 7, 8 or 9 and the median was 7 or lower.
- *Excluded*
  All other cases.

Since most indicators (nearly 77%) fell in the ‘accepted’ or ‘excluded’ category, a second questionnaire round, usually conducted in a Delphi study, was skipped. Indicators in the ‘accepted’ and ‘equivocal’ categories were further discussed in focus groups (step 3). The narratives to the open-ended question (i.e., additional quality indicators) were qualitatively analyzed. One researcher (MZ) read all narratives, clustered those with the same meaning and summarized them into additional quality indicators. Subsequently, these unique additional quality indicators were discussed in focus group interviews (step 3) with all panelists.

### Step 3. Focus groups

Three focus group meetings were organized with the primary aim to discuss and reach consensus on the follow-up of indicators (i.e., accepted or equivocal) that progressed after round 1. The focus groups were also used to discuss on possible overlap between indicators and the potential need to redefine or merge indicators. When prioritizing indicators, also the validity and feasibility of the indicators were taking into account based on the participants’ expertise and experiences with the delivery and receipt of ICU care. In addition, the results from the open-ended question (i.e., the additional quality indicators) were discussed. Moreover, reasons why indicators were ‘'excluded” were briefly discussed. The focus groups were organized for, respectively, intensivists, ICU nurses, and former ICU patients and relatives. We chose for homogenous groups to avoid possible dominance by participants or power/ dependence relationships in the group that could hinder an open discussion. All three focus groups were held in the hospital setting with MZ as the moderator and lasted between 53 and 131 min. The guide for the focus groups is included in Supplementary File 1. Conversations were recorded through note-taking and audio-recording.

### Step 4. Establishing a core set of indicators

The notes and audio-recordings from the focus groups were analyzed by MZ to order the reasons for excluding indicators, the number of (finally) accepted and excluded indicators, and the proposed revisions to indicators. Findings were translated into a consensus-based core set of indicators each provided with an operationalization. MZ, RV and HH categorized the final indicators into overarching domains.

## Results

### Characteristics of the expert panel

In round 1, 34 experts received a questionnaire survey of which 100% responded: 11 intensivists, 13 ICU nurses and 10 former ICU patients and relatives. Mean age of the ICU nurses was 43 years (min–max 25–59) with a mean work experience of 14 years (min–max 1–38), mean age of the intensivists was 44 year (min–max 39–58) with a mean work experience of 9 years (min–max 3–23), mean age of the patients and relatives is 54 years (min–max 34–66) with a mean length of stay in the ICU (or of their relative) of 2.3 days (min–max 0.5–5.5 days) (Table 1).

**Table 1:** Characteristics of panel experts

Characteristic	Intensivists	ICU nurses	Patients/relatives
Number, n	11	13	10
Sex, male (%)	73	23	50
Mean age, years (min–max)	44 (39–58)	43 (25–59)	54 (34–66)
Mean work experience, years (min–max)	9 (3–23)	14 (1–38)	NA
Working in an academic hospital (%)	55	46	NA
Mean length of stay, days (min–max)	NA	NA	2.3 (0.5–5.5)

*NA *Not applicable

The overall response rate for the three focus group discussions in round 2 was 68% (*n* = 9 intensivists, *n* = 9 ICU nurses, and *n* = 5 patient and relatives respectively). Reasons for non-response were time constrains and scheduling problems.

### Step 1. Narrowing of pre-existing overview of ICU quality indicators

The total list of 122 quality indicators was reduced to a list of 69 indicators. These indicators were categorized into seven domains:

1. Organization of care in the ICU (5 items)
2. Outcomes of ICU treatment (8 items)
3. Occurrence of complications and iatrogenic injury (33 items)
4. Learning from complications and incidents (6 items)
5. Functioning of individual healthcare professionals and teams (4 items)
6. Experiences of patients and relatives (5 items)
7. Patient reported outcomes after discharge (8 items)

The full list of indicators is included in Supplementary file 2.

### Step 2. Rated relevance of indicators and proposed additional indicators

Based on the relevance scores, 41 of the 69 indicators (59%) were excluded. Twelve (17%) were accepted and for 16 indicators (23%) there was no agreement (Fig. 1, Table 2 and Supplementary files 2 and 3).

**Table 2:** Overview of number and type of quality indicators (QIs) from Delphi step 2 to 4

**Domain**	**QIs in questionnaire (*n* = 69)**	**QIs discussed in focus groups (*n* = 38)**^a^	**QIs in final core set (*n* = 17)**
Organization of ICU care	5	2	1
Outcomes of ICU treatment	8	2	2
Occurrence of complications and iatrogenic injury	33	4	2
Learning from complications and incidents	6	6	1
Functioning of individual professionals and teams	4	5	3
Experiences of patients and relatives	5	3	3
Patient reported outcomes after discharge	8	8	5

^a^The 10 additionally identified quality indicators are not categorized into the domains

*QI* Quality Indicator, *ICU* Intensive Care Unit

Examples of immediately excluded indicators are nursing workload, length of ICU stay and duration of mechanical ventilation, ICU and hospital mortality, several complications (e.g. tracheostoma related problems, acute kidney injury, difficult intubation, pneumothorax during ICU admission, patients with hyper or hypo glycaemia) and several compliance indicators (e.g. compliance to sepsis guidelines, to the perioperative surgical checklist, and to hand hygiene guidelines).

A convincing majority of the panel considered twelve indicators as relevant, including internal audit, quality visitation (i.e. site visit by intensivists of other hospitals), conferences about complications, preventable adverse events and deaths, local incident reporting, complaints, critical incidents reported to a central supervising authority (e.g. the Health Inspectorate), experiences of former ICU patients, experiences of relatives (thought post ICU follow-up clinic and via questionnaire), and quality of life of former ICU patients (Supplementary files 2 and 3).

Sixteen indicators without agreement were ICU readmissions, Standardized Mortality Ratio (SMR), number of patients with severe sepsis, incidence of pressure ulcers, incidence of delirium, percentage of medication errors, team climate, compliance to the ‘Crew resource Management (CRM)’ principles (set of trained procedures for use in high-risk situations), safety culture, quality of life of relatives of former ICU survivors, and indicators regarding physical functioning (i.e., frailty, fatigue and physical problems of former patients), mental functioning (i.e., Post-Traumatic Stress Disorder (PTSD), Anxiety and depression of former patients) and cognitive functioning of former ICU patients (Supplementary files 2 and 3).

The analysis of narratives on proposed quality indicators led to ten additional quality indicators: mental wellbeing of ICU clinicians (burnout rates), indicators regarding information and treatment (i.e. percentage patients that received information about long term outcomes, percentage patients that received early mobilization on the ICU, percentage ICU patient discharged with a rehabilitation treatment plan, percentage patient with ICU-Acquired Weakness that received neurological consult, compliance to delirium prevention interventions), resilience of patients, proportionality of ICU treatment, socio-economic impact of ICU stay and cost-effectiveness of ICU care (Supplementary file 3). These ten additional indicators were subject of discussion in the focus groups.

### Step 3. Follow-up of indicators based on group discussion by panel members

Table 2 and Supplementary file 3 describe the follow-up of indicators based on the group discussion by panel members. After discussions panelists generally agreed to include four of the 12 ‘accepted’ indicators (i.e., quality visitation, complaints, experiences of former ICU patients and quality of life of former ICU patients) and eight of the 16 ‘equivocal’ indicators (i.e., SMR, incidence of pressure ulcers, incidence of delirium, team climate, CRM compliance, safety culture, quality of life of relatives of former ICU survivors and ICU readmissions) in the final core set. Eleven indicators were excluded after discussion, including, for example, the number of patients with severe sepsis (mainly based on the argument that sepsis is a diagnosis, not a quality indicator) and the percentage of medication errors (mainly because necessary data collection is not feasible in practice). One ‘accepted’ indicator, internal audit, was excluded after the focus groups, because panelists argued that it fully overlapped with the quality visitations by peers (both focus on the assessment of the organization and management of care). Of the ten additionally identified indicators, two were finally selected for the core set: socio-economic impact and cost-effectiveness of ICU care.

In addition, based on the input of the focus groups five indicators (i.e., preventable adverse events rates, local incident reporting, critical incidents reported to a central supervising authority (e.g. the Health Inspectorate), complication conferences and multidisciplinary complication conferences) were merged into one overarching indicator called ‘learning and improving from serious incidents’. This indicator describes the number of performed and documented quality improvement (Plan-Do-Check-Act, PDCA) cycles following the occurrence of a serious safety incident. The aim of this indicator was to switch the focus from measurement (counting incidents) to actual quality improvement. Furthermore, the two indicators regarding experiences of relatives (based on collecting experiences through post ICU clinic versus questionnaire) were combined in one indicator called ‘experiences of relatives’. Finally, six indicators (i.e., frailty, fatigue, physical problems, PTSD, anxiety and depression, and cognitive functioning of former ICU patients) were merged into an overarching indicator called ‘health problems of ICU survivors’.

In the focus groups, panelists addressed several reasons why indicators were excluded after the questionnaire. First, indicators were never used by the ICU management (e.g. nursing workload). Second, indicators were perceived as not meaningful for measuring quality, like several compliance measures (e.g. compliance to the perioperative surgical checklist). Third, indicators were not actionable or did not provide sufficient information for improvement because of their low incidence (e.g. line infections and ‘the number of patients whose ICU experiences were assessed’). Fourth, indicators were considered routine care (e.g., compliance rates regarding pain management protocols, the sepsis bundle, hand hygiene guidelines and perioperative surgical checklist). Panel members, particularly ICU clinicians, expressed that the registration of adherence to standard ICU care procedures were, therefore, not meaningful. Moreover, such registrations give a sense of being distrusted by supervising parties.

### Final core set of quality indicators

The Delphi method resulted in a final core set of 17 quality indicators, divided into four categories (Table 3):

1. Organization of ICU care
2. Outcomes of ICU care
3. Patient reported experience measures (PREMs)
4. Patient reported outcome measures (PROMs)

**Table 3:** Final core set of indicators for quality improvement in the ICU

	**Organization (structure and process measures)**	**Outcome measures**
Reported by Health Care Professional	Team climate Safety culture CRM-compliance Quality visitation^a^ Learning from and improving after serious incidents	SMR ICU-readmissions with 48 h Incidence of delirium Incidence of pressure ulcer
Reported by Patient and Relative	Experiences of former ICU patients Experiences of relatives Complaints	Quality of life of former ICU patients Quality of life of relatives Physical, mental and cognitive functioning of ICU survivors (e.g. fatigue, frailty, anxiety, depression, PTSD, loss of memory) Socio-economic impact of ICU stay Cost-effectiveness of ICU care (quality of life versus costs)

^a^Site visit by intensivists of other hospitals

*CRM* Crew Resource Management*, ICU* Intensive Care Unit*,* *PTSD* Post Traumatic Stress Disorder*, SMR* Standardized Mortality Ratio

The five indicators in the ‘organization of ICU’ domain indicate the availability of the organizational preconditions for quality and safety of care: safety culture, team climate (the ability of a team to learn and improve), adherence to CRM (principles related to teamwork and communication on the ICU), and points of organizational improvements based on a site visitation by intensivists (peers). In addition, a new process indicator ‘learning and improving after serious incidents’ was formulated addressing the learning capacity of ICUs.

Moreover, in the category ‘outcomes of ICU care’ four healthcare provider reported outcomes were selected: SMR, ICU readmissions (further specified to readmissions within 48 h), and incidence of pressure ulcer and delirium.

Indicators in the last two categories are PREMs (patient reported experiences measures) and PROMs (patient reported outcome measures), including physical, mental and cognitive functioning of ICU survivors, and socio-economic burden (e.g., work-related problems). Also included in the last category are ‘cost-effectiveness of ICU care calculated by the reported quality of life post ICU versus healthcare costs’ and ‘quality of life reported by relatives of former ICU patients’. All 17 core set indicators are operationalized in Supplementary file 4.

## Discussion

This Delphi study, including 34 ICU clinicians, ICU survivors and their relatives, resulted in a core set of 17 indicators for continuous improvement of quality of care in the ICU. The core set is short and comprehensive as it provides information about organizational preconditions to assure quality of care (e.g. safety culture and team climate with open communication and direct feedback), validated indicators for benchmarking (e.g. SMR and ICU readmissions), and patient and relative reported experiences and outcomes to improve ICU care from a patient and relative perspective.

Compliance indicators regarding several clinical procedures, for example adherence to the perioperative surgical checklist, sepsis protocol, pain protocol, were not selected in the core set based on the assumption that such clinical procedures are self-evident and part of the core business of ICU care. The use of such indicators by check-marking registration can be a temporary useful strategy, for example to monitor quality problems or the implementation of guidelines. However, it is regarded less useful if registered for each patient during each working shift [3]. Our study indicates that such indicators and related registration activities can reinforce ICU physician and nurse mistrust and fear of reprimand by management, and ultimately do not contribute to better quality of ICU care. In fact, it may even replace clinical reasoning (i.e., thinking and handling based on checklists and marking scores rather than actions driven by own clinical assessments and observations) [3]. By excluding compliance measures, the focus of quality improvement can be shifted to outcomes relevant for patients, including surviving rates, short- and long-term functioning, and quality of life.

In the process of prioritizing and selection, panelists emphasized that not quality measurement per se but improvement actions resulting from measurements are crucial to improve patient outcomes. Therefore, several labor-intensive measurements, including local incident reporting and record review on adverse events and preventable deaths, were prioritized less relevant and ultimately replaced by the new indicator called ‘learning from and improving after serious incidents’. These findings correspond with previous literature addressing a paradigm shift in healthcare quality improvement, namely using performance data and measures to stimulate direct quality improvement rather than for accountability and benchmarking purposes only [6]. For continuous quality improvement one should aim for solving quality problems to reduce adverse outcomes and improving quality of care by running PDCA-cycles on clinical themes of interest, instead of counting incidents. Making these PDCA-cycles transparent and accessible, gives clinicians from other ICUs the opportunity to learn from similar quality problems in their own setting and gives boards, regulators and administrators real valuable monitoring and steering information.

Apart from stimulating the learning capacity of the ICU, the selected core set of quality indicators is a valuable contribution to ICU quality improvement literature as it supports efforts to govern on a broad range of quality aspects (i.e., at organizational, health care (team) and patient level) using different sorts of information. This core set includes both quantitative indicators (e.g. SMR, ICU-readmissions, incidence of pressure sores) to benchmark performance over time or between ICUs, as well as qualitative, aggregate information (e.g. recommendations from the quality visitation by peers) to interpret the quantitative figures. Moreover, this core set strongly focusses on quality information based on experiences of former ICU patients and relatives, while other core sets were focused on outcome measures regarding the physical, mental and cognitive functioning of ICU survivors [10, 12]. Finally, this core set is relatively short and yet comprehensive. We found only one study that developed quality indicators for ICU care that was rather lengthy (*n* = 42 indicators) and focused on one specific patient group (i.e., traumatic brain injury) [7]. Panel experts in that study expressed anticipated administrative burden for ICU clinicians as the main barrier to operationalizing the core set. We believe that this core set of 17 indicators minimizes the risk of administrative burden and facilitates continuous quality improvement. Data on these indicators are currently collected in several ICU’s in the Netherlands and will help to evaluate this coreset in the near future.

### Limitations

The development of the core set should be seen in the light of several limitations. First, the selection of indicators is based on perceived added value for quality improvement and patient outcomes. In general, most of the used ICU quality indicators in daily practice have not been scientifically evaluated on reliability, validity and discriminability. This is also the case for the indicators in this core set. In hindsight, validated standards such as the QUALIFY-tool could have improved the systematic assessment of the indicators on relevance, scientific soundness and feasibility [17, 18]. Additionally, some indicators may not be actionable, because there is no or limited evidence for effective interventions to prevent delirium in the ICU [19], or to prevent or mitigate adverse long-term patient outcomes [20]. Second, the core set was selected before the COVID-19 pandemic; during ‘a normal ICU setting’. During the pandemic, the mental health of ICU clinicians emerged as an important quality topic not included in this core set. This shows that the core set should be dynamic and regularly updated to include new quality problems and developments in ICU care. Third, the indicators were selected by an expert panel whose priorities may be influenced by the country-specific context and healthcare system. Findings may correspond with international priorities but are not necessarily generalizable to other countries. Fourth, as this study was conducted in 2017, presented findings might seem less relevant in 2022. However, during these five years no fundamental changes have occurred with regard to the governance of quality of intensive care and related registrations. Although the nature and the consequences of inefficient quality registrations in the ICU have been studied since our Delphi was performed [3], suggested means to improve quality assessment and governance of intensive care remained limited. Therefore, we assume that this study still resembles the present perceptions of stakeholders on necessary quality indicators.

## Conclusions

ICU clinicians and former ICU patients and relatives selected a core set of indicators for governance of quality improvement in the ICU that is tailored to the needs of patients and relatives. This core set can contribute to a shift in governance style from measurement to effective quality improvement in the ICU. The effectiveness of this core set on patient outcomes and on reduced registration burden is currently being evaluated in eights ICUs in the Netherlands.

## Supplementary Information

**Additional file 1.**

**Additional file 2.**

**Additional file 3.**

**Additional file 4.**

## Data Availability

The raw datasets generated during and analysed during the current study are not publicly available because some parts of the data can breach participant’s and institutional privacy, but are available after de-identification from the corresponding author on reasonable request.

## Abbreviations

ICU: Intensive Care Unit
PROM: Patient reported outcome measure
PREM: Patient reported experience measure
FCIC: Family and Patient Centered Intensive Care
Min: Minimum
Max: Maximum
NA: Not Applicable
SMR: Standardized Mortality Ratio
CRM: Crew Resource Management
PTSD: Post-Traumatic Stress Disorder
QI: Quality Indicator
PDCA: Plan-Do-Check-Act
