# Supplementary material for: Putting measurement on a diet: development of a core set of indicators for quality improvement in the ICU using a Delphi method
Source: BMC Health Serv Res. 2022 Jul 5;22:869. doi: 10.1186/s12913-022-08236-3 (PMC9255461; doi:10.1186/s12913-022-08236-3)
Supplement: Supplementary file 1 — Additional file 1. [file 12913_2022_8236_MOESM1_ESM.docx]

**Supplementary File 1. Guide for focus group interviews**

- What are important outcomes for you? When is the ICU treatment successful? *[This was the central question in the meeting with former patients and relatives]*

**Short explanation about the questionnaire and the analysis of data.*

- In total, [X] indicators were excluded based on the questionnaire results *[list of excluded indicators was presented to the panel]*. Can you give some reasons why certain indicators were excluded?

- There are [X] indicators that were accepted based on the questionnaire results *[list of accepted indicators was presented to the panel]*. Moreover, there is no consensus about [X] indicators *[list of equivocal indicators was presented to the panel]*. Can you indicate per indicator which indicator should be included in the core set and which indicator not? And why?

- Are there subjects not included in any quality indicator/registration at all, but are important to improve quality of care and outcomes?

- Are there indicators that need to be redefined, merged or further specified?
