# Supplementary material for: Putting measurement on a diet: development of a core set of indicators for quality improvement in the ICU using a Delphi method
Source: BMC Health Serv Res. 2022 Jul 5;22:869. doi: 10.1186/s12913-022-08236-3 (PMC9255461; doi:10.1186/s12913-022-08236-3)
Supplement: Supplementary file 2 — Additional file 2. [file 12913_2022_8236_MOESM2_ESM.docx]

**Supplementary File 2. Questionnaire format and results per quality indicator**

**FORMAT**

In this questionnaire, quality indicators that are registered in the ICU for quality improvement, governance and accountability are listed. The quality indicators can be divided into seven domains:

- Organization of care in the ICU (5 items)
- Outcomes of ICU treatment (8 items)
- Occurrence of complications and iatrogenic injury (33 items)
- Learning from complications and incidents (6 items)
- Functioning of individual healthcare professionals and teams (4 items)
- Experiences of patients and relatives (5 items)
- Patient reported outcomes after discharge (8 items)

1. **Please rate on a scale from 1 to 9 to what extent the indicator and related measurement data is useful for improving clinical performance, health outcomes and patient experiences (1 = totally not relevant, 9 = very relevant).**
2. **Which subjects or quality indicators do you think are relevant to improve patient care and outcomes but were not mentioned in the questionnaire?**

**RESULTS**

*Selection of quality indicators was based on the rated relevance scores by intensivists, nurses, former patients and relatives:

*Accepted*
A convincing majority of participants considered the parameter relevant: at least 70% of participants scored 7, 8 or 9 and the median was at least 8.

*Equivocal*Extremely skewed distribution: at least 30% of participants scored 1, 2 or 3 and at least 30% of participants scored 7, 8 or 9; or somewhat skewed distribution: at least 70% of participants scored 7, 8 or 9 and the median was 7 or lower.

*Exclusion*

All other cases.

1. *Organization of care in the ICU*

| **Quality indicator** | **Operationalization** | **Median** | **Lowest tertile (%)** | **Highest tertile (%)** |
| --- | --- | --- | --- | --- |
| Nursing workload | Nursing workload for efficient planning of nurses in the ICU measured with NAS/ TISS | 7 | 8.8 | 58.8 |
| Quality patient record | Quality of the patient record is adequate (yes/no)? If not, what are improvements? | 5 | 26.5 | 32.4 |
| Internal audit | Internal audit (interviews, observations, document study) to study the compliance to laws, guidelines and protocols. Conclusions of internal audits are recommendations and points of improvement. | 8 | 2.9 | 79.4 |
| JCI accreditation | Accreditation by auditors of the Joint Commission International to assess the compliance to several quality and patient safety guidelines, interventions and protocols. | 5 | 5.9 | 41.2 |
| Quality visitation | Quality assessment site visit by intensivists of other hospitals who are organized in the Dutch Society of Intensive Care (NVIC), resulting in conclusions and recommendations regarding leadership, medical and nursing staffing, availability of protocols, etc. | 8 | 0 | 82.4 |

*2. Outcomes of ICU treatment*

| **Quality indicator** | **Operationalization** | **Median** | **Lowest tertile (%)** | **Highest tertile (%)** |
| --- | --- | --- | --- | --- |
| Length of ICU stay | Mean length of stay on the ICU. | 5 | 23.5 | 47.1 |
| Mechanical ventilation | Mean duration of mechanical ventilation of patients on the ICU. | 7 | 14.7 | 52.9 |
| ICU readmission | Percentage patients who are readmitted to the ICU within the same hospital admission. | 7 | 2.9 | 76.5 |
| ICU mortality | Percentage of patients who died on the ICU. | 7 | 17.6 | 52.9 |
| Hospital mortality | Percentage of patients who died during hospital stay. | 6 | 14.7 | 50.0 |
| Standardized Mortality Ratio (SMR) | Ratio between the observed number of deaths and the number of deaths would be expected, based on prediction models correcting for e.g. age, sex and severity of illness (APACHE II, SAPS II of APACHE IV). | 7 | 0 | 76.5 |
| Sequential Organ Failure Assessment (SOFA) | Sequential Organ Failure Assessment (SOFA) score quantifies the number and severity of organ failure. | 7 | 5.9 | 55.9 |
| Patient transfer | Percentage of patients who were transferred to other hospitals. | 5 | 29.4 | 20.6 |

1. *Occurrence of complications and iatrogenic injury*

| **Quality indicator** | **Operationalization** | **Median** | **Lowest tertile (%)** | **Highest tertile (%)** |
| --- | --- | --- | --- | --- |
| Severe sepsis (compliance) | Compliance screening for severe sepsis (%). | 7 | 20.6 | 58.8 |
| Resuscitation bundle severe sepsis | Compliance to the resuscitation bundle for severe sepsis (%). | 7 | 11.8 | 67.6 |
| Severe sepsis | Number of patients with severe sepsis. | 6 | 32.4 | 47.1 |
| Time in situ of intravenous lines | Time in situ of intravenous lines (amount and sort of lines, number of minutes and days in situ) | 6 | 17.6 | 44.1 |
| Insertion central venous and arterial catheters | Compliance to protocols for insertion procedure (%). | 7 | 17.6 | 52.9 |
| Daily control central venous and arterial catheters | Compliance to protocol for daily control of venous and arterial catheters (%). | 8 | 5.9 | 61.8 |
| Indication central venous and arterial catheters | Compliance to protocol for daily control of indication for venous and arterial catheters (%). | 7 | 5.9 | 55.9 |
| Line sepsis | Rate of line sepsis per 1000 catheter days. | 7 | 5.9 | 67.6 |
| Hand hygiene | Compliance to hand hygiene guidelines to prevent infections (%). | 7 | 8.8 | 61.8 |
| MRSA | Percentage MRSA (Methicillin-resistant Staphylococcus aureus) | 5 | 11.8 | 29.4 |
| Incidence pressure ulcers | Percentage ICU acquired pressure ulcers. | 7 | 2.9 | 73.5 |
| Screening delirium | Compliance screening for delirium (%). | 7 | 17.6 | 58.8 |
| Incidence delirium | Percentage patients with delirium (positive outcome screening instrument). | 7 | 5.9 | 70.6 |
| Pain | Number of pain measurements carried out (compliance measurement) | 6 | 29.4 | 47.1 |
| Pain | Used validated instrument to measure pain (VAS, NRS, BPS, CIA of CPOT). | 7 | 8.8 | 67.6 |
| Pain | Pain score. | 7 | 5.9 | 67.6 |
| Glycaemia | Percentage patients with hyper or hypo glycaemia (> 8.0 mmol/l and < 2.2 mmol/l) | 7 | 8.8 | 52.9 |
| Hypo glycaemia | Median duration (hours) between hypo glycaemia determination and new measurement | 6 | 20.6 | 50.0 |
| Hypo glycaemia | Median duration (hours) between hypo glycaemia determination and glucose value > 2.2 mmol/l | 7 | 5.9 | 58.8 |
| High risk medication | Compliance to high risk medication guidelines (%). | 7 | 8.8 | 61.8 |
| Malnutrition | The amount of severely malnourished adult patients with adequate protein intake at admission day 4 compared to the amount of severely malnourished adult patients on day 5. | 6 | 23.5 | 47.1 |
| Malnutrition | Measuring energy intake and consumption (calorimetry) | 6 | 14.7 | 47.1 |
| Medication verification at admission | Compliance to medication verification guidelines (%). | 7 | 8.8 | 58.8 |
| Pevention medication errors | Adherence to policy to prevent medication errors. | 7 | 14.7 | 64.7 |
| Medication errors | Percentage medication errors. | 7 | 11.8 | 70.6 |
| Re-intubation | Number of re-intubations after detubation. | 7 | 5.9 | 67.6 |
| Pneumothorax | Percentage ICU acquired pneumothorax. | 6 | 20.6 | 38.2 |
| ICU acquired weakness | Percentage ICU acquired weakness | 7 | 14.7 | 61.8 |
| Difficult intubation | Percentage patients with difficult intubation and/or airway related problems | 6 | 23.5 | 38.2 |
| Tracheostomy related problems | Percentage patients with problems/complications with tracheostomy or tracheal cannula | 6 | 20.6 | 38.2 |
| Acute kidney injury | Percentage patients without previous renal insufficiency (creatinine <171 umol/l) who reach a SOFA score of 3 or higher during ICU admission | 7 | 11.8 | 52.9 |
| Quality of sleep | Sleep score given by patients. | 7 | 2.9 | 52.9 |
| Physical restraints | Number of patients with physical restraints | 7 | 20.6 | 55.9 |

1. *Learning from complications and incidents*

| **Quality indicator** | **Operationalization** | **Median** | **Lowest tertile (%)** | **Highest tertile (%)** |
| --- | --- | --- | --- | --- |
| Complication conferences | Frequency and nature of discussed complications and formulated points of improvement. | 8 | 0 | 94.1 |
| Multidisciplinary complication conferences | Frequency and nature of discussed complications and formulated point of improvement. | 8 | 2.9 | 94.1 |
| Preventable adverse events and deaths | Frequency and nature of adverse events among deceased patients. | 8 | 0 | 97.1 |
| Local incident reporting | Frequency and nature of reported incidents and related points of improvement. | 8 | 2.9 | 79.4 |
| Complaints | Frequency and nature of reported complaints and related points of improvement. | 8 | 2.9 | 9.4 |
| Critical incidents reported to a central supervising authority | Frequency and nature of critical incidents reported to a central supervising authority (i.e. the Health Inspectorate). | 8 | 0 | 97.1 |

1. *Functioning of individual healthcare professionals and teams*

| **Quality indicator** | **Operationalization** | **Median** | **Lowest tertile (%)** | **Highest tertile (%)** |
| --- | --- | --- | --- | --- |
| Team climate | Frequency and reported outcomes of assessing the functioning of and collaboration within a team of ICU professionals (for example using the Team Climate Inventory). | 7 | 5.9 | 82.4 |
| Crew Resource Management principles (CRM) | Compliance to CRM principles for improving for teamwork and safety culture (observations). | 7 | 2.9 | 73.5 |
| Perioperative surgical checklist | Compliance to the perioperative surgical checklist (registration in medical record). | 7 | 5.9 | 61.8 |
| Safety culture | Frequency and reported outcomes of assessing safety culture in the ICU (for example, using the Safety Attitudes Questionnaire). | 7 | 2.9 | 73.5 |

1. *Experiences of patients and relatives*

| **Quality indicator** | **Operationalization** | **Median** | **Lowest tertile (%)** | **Highest tertile (%)** |
| --- | --- | --- | --- | --- |
| Experiences of relatives (questionnaire) | Experiences of relatives, including subjects as treatment, information provision, communication, support and privacy, measured with the Consumer Quality Index (CQI). | 8 | 0 | 76.5 |
| Experiences of former ICU patients (post ICU clinic) | Experiences with ICU treatment and recommendations for quality improvement of former patients based on conversations in the post ICU clinic. | 8 | 2.9 | 85.3 |
| Experiences of relatives (post ICU clinic) | Experiences with ICU treatment and recommendations for quality improvement of relatives of former patients based on conversations in the post ICU clinic. | 8 | 0 | 79.4 |
| Experiences of former ICU patients | Number of patients of which experiences were measured and implemented quality improvements based on their feedback. | 8 | 0 | 64.7 |
| Experiences of relatives of former ICU patients | Number of relatives of which experiences were measured and implemented quality improvements based on their feedback | 7 | 0 | 64.7 |

1. *Patient reported outcomes after discharge*

| **Quality indicator** | **Operationalization** | **Median** | **Lowest tertile (%)** | **Highest tertile (%)** |
| --- | --- | --- | --- | --- |
| Quality of life of former patients | Quality of Life questionnaire (e.g. SF-36 /EQ-5D) | 8 | 0 | 91.2 |
| Quality of life of relatives | Quality of Life questionnaire (e.g. SF-36 /EQ-5D) | 7 | 8.8 | 76.5 |
| Frailty of former patients | Degree of frailty (Clinical Frailty Scale). | 7 | 0 | 79.4 |
| Fatigue of former patients | Degree of fatigue (Checklist Individual Strength). | 7 | 2.9 | 73.5 |
| Cognitive functioning of former patients | Score for Cognitive Functioning (Cognitive Functioning Questionnaire). | 7 | 2.9 | 73.5 |
| Post-Traumatic Stress Disorder (PTSD) among former patients | Post-Traumatic Stress Disorder questionnaire (IES-R). | 7 | 2.9 | 76.5 |
| Anxiety and depression of former patients | Anxiety and depression questionnaire (HADS). | 7 | 2.9 | 76.5 |
| Physical problems of former patients | Frequency of physical problems post ICU. | 7 | 2.9 | 76.5 |
