# Supplementary material for: Putting measurement on a diet: development of a core set of indicators for quality improvement in the ICU using a Delphi method
Source: BMC Health Serv Res. 2022 Jul 5;22:869. doi: 10.1186/s12913-022-08236-3 (PMC9255461; doi:10.1186/s12913-022-08236-3)
Supplement: Supplementary file 3 — Additional file 3. [file 12913_2022_8236_MOESM3_ESM.docx]

**Supplementary File 3. Flow of quality indicator selection through Delphi step 2-4**

**RESULTS QUESTIONNAIRE (STEP 2)**

**Accepted QIs (n=12):**

internal audit

quality visitation

conferences about complications

multidisciplinary conferences about complications

preventable adverse events and deaths

incident reporting

complaints

critical incidents reported to the Inspectorate

experiences of former ICU patients

experiences of relatives (post ICU clinic)

experiences of relatives (questionnaire)

quality of life of former ICU patients

**Equivocal QIs (n=16):**

ICU readmissions

Standardized Mortality Ratio (SMR)

number of patients with severe sepsis

incidence pressure ulcers

incidence delirium

percentage medication errors

team climate

compliance to the ‘Crew resource Management (CRM)’ principles

safety culture

quality of life of relatives of former ICU survivors

frailty

fatigue

physical problems

Post-Traumatic Stress Disorder (PTSD),

anxiety and depression

cognitive functioning of former ICU patients

**Additional QIs (n=10):**

mental wellbeing of ICU professionals (burnout rates)

percentage patients that received information about long term outcomes

percentage patients that received early mobilization on the ICU

percentage ICU patient discharged with a revalidation treatment plan

percentage patient with ICU-Acquired Weakness that received neurological consult

compliance to delirium prevention interventions

resilience of patients

proportionality of ICU treatment

socio-economic impact of ICU stay

cost-effectiveness of ICU care.

**RESULTS FOCUS GROUPS (STEP 3)**

**Of the 12 accepted QIs from the questionnaire…**

***accepted (n=4):**

quality visitation

complaints

experiences of former ICU patients

quality of life of former ICU patients

***merged into one accepted indicator called ‘Learning from and improving after serious incidents’ (n=5):**

conferences about complications

multidisciplinary conferences about complications

preventable adverse events and deaths

incident reporting

critical incidents reported to the Inspectorate

***merged into one accepted indicator called ‘Experiences of relatives’ (n=2):**

experiences of relatives (post ICU clinic)

experiences of relatives (questionnaire)

***excluded (n=1):**

internal audit

**Of the 16 equivocal QIs from the questionnaire…**

***accepted (n=8):**

Standardized Mortality Ratio (SMR)

incidence pressure ulcers

incidence delirium

team climate

compliance to the ‘Crew resource Management (CRM)’ principles

safety culture

quality of life of relatives of former ICU survivors

ICU readmissions

***merged into one accepted indicator called ‘****Physical, mental and cognitive functioning of IC survivors’ (n=6):**

frailty

fatigue

physical problems

Post-Traumatic Stress Disorder (PTSD)

anxiety and depression

cognitive functioning of former ICU patients

***excluded (n=2):**

number of patients with severe sepsis

percentage medication errors

**Of the 10 additional QIs from the questionnaire…**

***accepted (n=2):**

cost-effectiveness of ICU care

socio-economic impact of IC stay

***excluded (n=8):**

mental wellbeing of ICU professionals (burnout rates)

percentage patients that received information about long term outcomes

percentage patients that received early mobilization on the ICU

percentage ICU patient discharged with a revalidation treatment plan

percentage patient with ICU-Acquired Weakness that received neurological consult

compliance to delirium prevention interventions

resilience of patients

proportionality of ICU treatment

**THE ESTABLISHED CORE SET OF 17 ICU QUALITY INDICATORS (STEP 4)**

team climate

quality visitation

complaints

experiences of former ICU patients

experiences of relatives

quality of life of former ICU patients

quality of life of relatives

Standardized Mortality Ratio (SMR)

incidence pressure ulcers

incidence delirium

compliance to the ‘Crew resource Management (CRM)’ principles

safety culture

ICU readmissions within 48 hours

physical, mental and cognitive functioning of IC survivors

learning and improving after serious incidents

cost-effectiveness of ICU care

socio-economic impact of IC stay
