# Supplementary material for: Putting measurement on a diet: development of a core set of indicators for quality improvement in the ICU using a Delphi method
Source: BMC Health Serv Res. 2022 Jul 5;22:869. doi: 10.1186/s12913-022-08236-3 (PMC9255461; doi:10.1186/s12913-022-08236-3)
Supplement: Supplementary file 4 — Additional file 4. [file 12913_2022_8236_MOESM4_ESM.docx]

**Supplementary File 4. Operationalization of the 17 quality indicators included in the core set**

| **ORGANISATION OF ICU CARE** | |
| --- | --- |
| **Quality indicator** | **Operationalization** |
| Quality visitation | Quality assessment site visit performed by intensivists of other hospitals who are organized in the Netherlands Association for Intensive Care (NVIC), resulting in conclusions and recommendations regarding clinical and organizational aspects (e.g., leadership, medical and nursing staffing, availability of protocols). |
| Team climate | Frequency and reported outcomes of assessing the functioning of and collaboration within a team of ICU professionals (for example using the Team Climate Inventory). |
| Safety culture | Frequency and reported outcomes of assessing safety culture in the ICU (for example, using the Safety Attitudes Questionnaire). |
| Crew Resource Management (CRM) compliance | Compliance to the CRM principles for improving for teamwork and safety culture. |
| Learning from and improving after serious incidents | The number of performed and documented quality improvement (Plan-Do-Check-Act) cycles following the occurrence of a serious safety incident. |
| **OUTCOMES OF ICU CARE** | |
| **Quality indicator** | **Operationalization** |
| ICU Standardized Mortality Ratio (SMR) | Ratio between the observed number of deaths and the number of deaths would be expected, based on prediction models correcting (for example) on age, sex and severity of illness (APACHE II, SAPS II of APACHE IV). |
| ICU readmissions within 48 hours | Percentage patients who are readmitted to the ICU within 48 hours. |
| Incidence of pressure ulcer | Percentage ICU acquired pressure ulcer (stage 3 or 4). |
| Incidence of delirium | Percentage patients with delirium (positive outcome screening instrument) measured with, for example, the Confusion Assessment Method (CAM). |
| **Patient-reported experience measures (PREM)** | |
| **Quality indicator** | **Operationalization** |
| Patient-reported experiences | Reported experiences and recommendations for quality improvement of former ICU patients, measured with surveys or during post-ICU clinic visits. |
| Relative-reported experiences | Reported experiences and recommendations for quality improvement of relatives of ICU patients, measured with surveys or during post-ICU clinic visits. |
| Complaints | Frequency, number and nature of reported complaints by ICU patients and relatives, and related points of improvement. |
| **Patient-reported outcomes measures (PROM)** | |
| **Quality indicator** | **Operationalization** |
| Quality of life of former ICU patients | Self-reported quality of life of former ICU patients (for example, measured with the Short Form Survey (SF-36) or the EQ-5D). |
| Quality of life of relatives | Self-reported quality of life of relatives of former ICU patients (for example, measured with the Short Form Survey (SF-36) or the EQ-5D). |
| Health problems of ICU survivors | Occurrence of physical, mental and cognitive problems measured by self-reported screening instruments on frailty, fatigue, pain, muscle weakness, symptoms of post-traumatic stress disorder (PTSD), anxiety and depression. |
| Socioeconomic impact of ICU stay | Proportion of ICU survivors who are fully/partly able to return to work post ICU. |
| Cost-effectiveness of ICU care | Ratio self-reported quality of life of former ICU patients versus related ICU costs. |
